# AtGAP1 Promotes the Resistance to *Pseudomonas syringae* pv. *tomato* DC3000 by Regulating Cell-Wall Thickness and Stomatal Aperture in Arabidopsis

**DOI:** 10.3390/ijms23147540

**Published:** 2022-07-07

**Authors:** Sau-Shan Cheng, Yee-Shan Ku, Ming-Yan Cheung, Hon-Ming Lam

**Affiliations:** Centre for Soybean Research of the State Key Laboratory of Agrobiotechnology, School of Life Sciences, The Chinese University of Hong Kong, Hong Kong, China; chengsaushan@yahoo.com (S.-S.C.); cheungmy@cuhk.edu.hk (M.-Y.C.)

**Keywords:** GTPase activating protein (GAP), GTP, pathogen resistance, cell wall, stomatal aperture

## Abstract

GTP is an important signaling molecule involved in the growth, development, and stress adaptability of plants. The functions are mediated via binding to GTPases which are in turn regulated by GTPase-activating proteins (GAPs). Satellite reports have suggested the positive roles of GAPs in regulating ABA signaling and pathogen resistance in plants. However, the molecular mechanisms that bring forth the pathogen resistance have remained unclear. In this study, we demonstrated that the expression of *AtGAP1* was inducible by *Pseudomonas syringae* pv. *tomato* DC3000 (*Pst* DC3000). The overexpression of *AtGAP1* in Arabidopsis promoted the expression of *PR1* and the resistance to *Pst* DC3000. Proteomic analyses revealed the enhanced accumulation of cell-wall-modifying proteins as a result of *AtGAP1* overexpression. By microscopic analyses, we showed that the overexpression of *AtGAP1* resulted in increased thickness of the mesophyll cell wall and reduced stomatal aperture, which are effective strategies for restricting the entry of foliar pathogens. Altogether, we demonstrated that AtGAP1 increases the resistance to *Pst* DC3000 in Arabidopsis by promoting cellular strategies that restrict the entry of pathogens into the cells. These results point to a future direction for studying the modes of action of GAPs in regulating plant cell structures and disease resistance.

## 1. Introduction

GTPase-activating proteins (GAPs) are regulators of GTP-binding proteins (G-proteins) for controlling cellular signals. GAPs stimulate the GTPase activity of G-proteins by converting them between the active GTP-bound form and inactive GDP-bound form [1,2]. GAPs are involved in various stress responses in plants, fungi, insects, and mammals [3,4,5,6,7,8]. G-proteins have been suggested as the molecular switch that regulates a wide variety of plant processes such as defense [9,10,11], growth and development [12,13], and phytohormone responses [14,15]. One of the well-studied GAP proteins, the glucose-regulated GTPase-accelerating protein (AtRGS1) in Arabidopsis, was reported to regulate sugar, abscisic acid (ABA), and drought stress signals [16,17]. The homolog of AtRGS1 in mulberry, MaRGS, was reported to be a negative regulator of salt stress responses [8]. 

GAPs target the heteromeric G-protein α-subunit to regulate bacterial growth and disease susceptibility in plants [18,19]. In rice, OsGAP1 (NCBI accession number: XP_015627601) was previously reported to be involved in both biotic and abiotic stress responses [9,20,21]. In rice, the expression of *OsGAP1* was induced upon wounding [20]. It was suggested that the increased level of *OsGAP1* expression upon wounding helps trigger plant defense responses for better survivorship against infections by pathogens, including *Xanthomonas oryzae* pv. *oryzae* (*Xoo*) and *Pseudomonas syringae* pv. *tomato* DC3000 (*Pst* DC3000) [9,20]. OsGAP1 contains a C2 domain that enables its binding to phospholipids [20]. In addition, the protein-interacting partner of OsGAP1, an unconventional G-protein (OsYchF1), was shown to be a negative regulator of both salinity stress and pathogen infection in Arabidopsis [9,20,21,22]. It is hypothesized that OsGAP1 helps trigger plant defense responses by converting OsYchF1 into its inactive form by activating its GTPase and ATPase activities [9,20,21]. Additionally, OsGAP1 competes with 26S rRNA to bind OsYchF1 at its TGS domain [9,21]. It was proposed that OsGAP1 regulates the activity of OsYchF1 by translocating OsYchF1 from the cytosol when under normal stress-free conditions to the plasma membrane upon wounding [9,21], through its interactions with OsYchF1 [9]. Under salinity stress, both OsGAP1 and OsYchF1 were localized in the cytosol [21].

G-proteins are involved in the formation of both secondary cell wall and primary cell wall in Arabidopsis [23,24,25,26,27]. Small GTPases have been reported to regulate cell wall deposition [28,29]. For example, RabG3b is a GTP-binding protein regulating tracheary element (TE) differentiation, which is associated with secondary-cell-wall deposition [30]. On the other hand, Rho GTPase ROP11 recruits a plant-specific microtubule-binding protein (MIDD1) to induce the local disassembly of cortical microtubules [31], which are associated with cellulose microfibril deposition for secondary-cell-wall thickening [32]. GTPases also regulate the formation of primary cell wall, which is not lignified and is thinner compared to secondary cell wall [28]. For example, the Rab GTPases Rab11 and RabA4d regulate the membrane trafficking for the delivery of cell-wall materials [33,34]. Previous reports also suggested the possible involvement of a G-protein subunit or G-protein receptor-like module in regulating the trafficking of cellulose synthase from the Golgi apparatus to the plasma membrane for cellulose production [25,27].

The homologous proteins OsGAP1 and AtGAP1 (AT3G17980) share a 59% identity in amino-acid sequences. AtGAP1 is also described as C2-domain ABA-related (CAR4) protein and was reported to be involved in abscisic acid (ABA) signaling [35,36]. AtGAP1 (CAR4) mediates the transient interaction of the pyrabactin-resistance 1/PYR1-like (PYR/PYL) regulatory components of ABA receptors (RCAR) during their recruitment to the plasma membrane in a calcium-dependent manner, and thus affects the subcellular localization of PYR/PYLs [35,36]. AtGAP1 (CAR4) acts as a positive regulator of ABA signaling by promoting the interaction of PYR/PYLs with clade-A protein phosphatases type 2C (PP2Cs) and inhibits the PP2C-mediated dephosphorylation of ABA-activated sucrose nonfermenting 1-related protein kinases subfamily 2 (SnRK2s). Activated SnRK2s go on to phosphorylate ABF transcription factors for the transcription of ABA-responsive genes in the cell [37,38,39]. *car* mutants showed reduced sensitivity to both ABA-mediated inhibitions of primary root and salt-induced inhibition of lateral root growth. It is hypothesized that environmental stresses might induce oscillation of the cellular Ca^2+^ level, and the CAR proteins might mediate the crosstalk between ABA and Ca^2+^ signaling in cells [35,36].

The restriction of pathogen entry is the first line of plant defense. Stomata are an important gateway for the entry of foliar bacterial pathogens [40]. Foliar bacterial infection is more efficient in high humidity which favors stomatal opening [40]. Upon *Pseudomonas syringae* infection, flg22, the epitope on the flagellum of the bacterial cell and the pathogen-associated molecular pattern (PAMP), is recognized by FLS2, the receptor on leaf epidermal cells. FLS2 then triggers stomatal closure mediated by salicylic acid (SA) and ABA to restrict the entry of the bacterial cells [41]. If they manage to pass through the stomata, the bacterial cells can get into intercellular air spaces in the spongy parenchyma and infect the surrounding cells [42]. Pathogens secrete cell wall-degrading enzymes (CWDEs) to facilitate the infection [43,44,45]. In Arabidopsis, INFLORESCENCE DEFICIENT IN ABSCISSION (IDA)-like 6 (IDL6)-HAESA (HAE)/HAESA-LIKE2 (HSL2) was reported to promote pectin degradation to facilitate *Pst* DC3000 infection [46]. It was suggested that *Pst* DC3000 might be able to manipulate the IDL6-HAE/HSL2-ADP2 signaling pathway to promote the infection of Arabidopsis leaves [46]. The cell wall is a mechanical barrier at the front line of preventing the entry of pathogens into the cells [47,48,49]. In Arabidopsis, the mutation of *WAT1* (*Walls are thin 1*) led to a reduction in secondary-cell-wall thickness [50] and improved resistance to pathogens of vascular plants including bacteria and fungi [51].

OsGAP1 was reported as a positive regulator of pathogen resistance [9,20] while AtGAP1 (CAR4) was reported as a regulator of ABA signaling [35,36]. However, the role of AtGAP1 upon pathogen infection has remained unclear. In this study, we generated *AtAGP1*-overexpressing Arabidopsis plants and showed that *AtGAP1* overexpression enhanced the resistance to *Pst* DC3000 infection by increasing the accumulation of cell-wall-modification-related proteins prior to pathogen infection, as well as increasing the cell wall thickness of mesophyll cells and reducing the stomatal aperture of Arabidopsis leaves.

## 2. Results

### 2.1. AtGAP1 Is a Positive Regulator of Pst DC3000 Resistance in Arabidopsis

**Figure 1:**
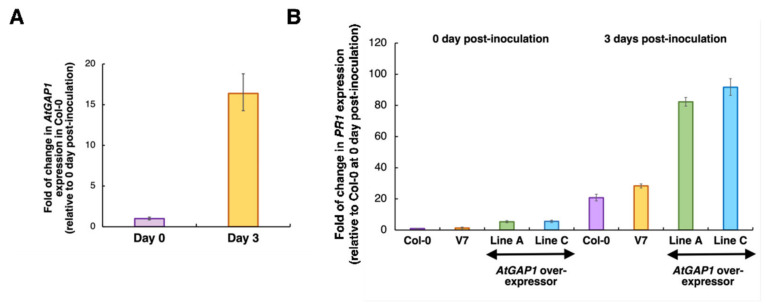
*AtGAP1* is inducible in Arabidopsis by pathogen (*Pst* DC3000) inoculation and *AtGAP1*-overexpressors are more effective in activating the pathogen-resistance marker gene, *PR1*, than wild type or the empty vector control. The rosette leaves of five-week-old wild type Arabidopsis (Col-0), empty vector control (V7), and *AtGAP1*-overexpressors (independent lines A and C) were inoculated with *Pst* DC3000. Total RNA was extracted from the aerial part of the plants. (**A**) *AtGAP1* expression was induced in Col-0 at 3 days post-inoculation (Day 3) with *Pst* DC3000, compared to Day 0. (**B**) The relative expressions of *PR1* in wild type (Col-0), V7 (empty vector control), and *AtGAP1*-overexpressors (lines A and C) at 0 and 3 d post-inoculation (dpi). Gene expression levels were normalized to 0 dpi levels of Col-0, using *ACT7* (AT5G09810) as the reference gene [54], by the 2^−ΔΔCt^ method [55]. Three plants of each line were pooled as one sample for total RNA extraction and expression analysis. For each sample, three technical repeats of the RT-qPCR were performed. Error bars represent the standard errors of the three technical repeats. A similar expression trend was observed in another biological repeat (Figures S3 and S4).

AtGAP1 shares a 59% identity in amino acid sequence with OsGAP1 (Figure S1) [52], which was reported to be a positive regulator of defense against pathogen infection in rice [9,20]. To test if AtGAP1 has a similar role in Arabidopsis, five-week-old plants, including wild type (Col-0), empty vector control (V7), and *AtGAP1*-overexpressors (independent lines A and C) were inoculated with *Pst* DC3000 [20]. The overexpression of *AtGAP1* in the transgenic lines was verified by RT-qPCR (Figure S2). The expression of *AtGAP1* was found to be inducible in the wild type by *Pst* DC3000 inoculation (Figure 1A). At both 0 and 3 days post-inoculation (dpi), *AtGAP1-*overexpressors had higher expression levels of the defense marker gene *Pathogenesis-Related 1* (*PR1*) [53] (Figure 1B).

**Figure 2:**
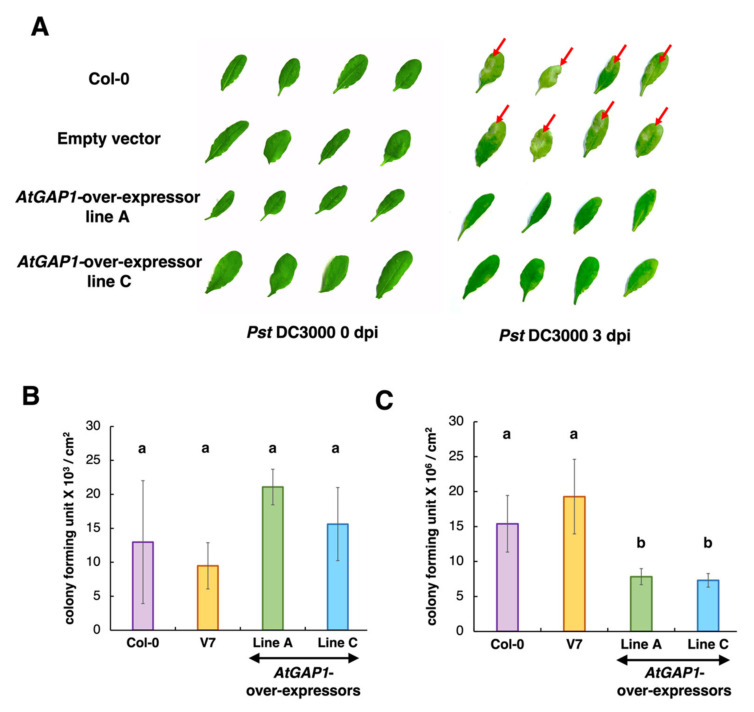
*AtGAP1*-overexpressing Arabidopsis showed a more resistant phenotype to *Pst* DC3000 than wild type and the empty vector control. The rosette leaves of five-week-old Arabidopsis plants, including the wild type (Col-0), empty vector control (V7), and *AtGAP1*-overexpressors (independent lines A and C) were inoculated with *Pst* DC3000. (**A**) The *Pst* DC3000-induced lesions in the rosette leaves at 0 and 3 days post-inoculation (dpi), indicated by red arrows. There were no visible lesions on the leaves of *AtGAP1*-overexpressors at 3 dpi. (**B**) Pathogen titers of the inoculated rosette leaves expressed in colony-forming units per cm^2^ of the leaf surface area at 0 dpi. (**C**) Pathogen titers of the inoculated rosette leaves at 3 dpi. (**B**) and (**C**) Different letters indicate significant differences at *p* < 0.05, using one-way ANOVA followed by post-hoc Tukey honestly significant difference (HSD) test. The results represent the average of three biological replicates. In each biological replicate, three individual plants were inoculated with *Pst* DC3000 for the pathogen titer analysis. Error bar: standard error; *n* = 9 plants from 3 biological replicates.

Since *PR1*, the defense marker gene, was more highly induced in the *AtGAP1*-overexpressors after *Pst* DC3000 inoculation, we tested if this translated into greater resistance of the overexpressor lines against the pathogen (Figure 2). Three days after *Pst* DC3000 inoculation, lesions were observed at the sites of inoculation in the rosette leaves of wild type and empty vector control but not in the *AtGAP1-*overexpressors (Figure 2A). In addition, at 3 dpi, the wild type and empty vector control plants had higher pathogen titers compared to the *AtGAP1-*overexpressors (Figure 2B,C). These results suggest that AtGAP1 is a positive regulator of the resistance against the pathogen, *Pst* DC3000, in Arabidopsis.

### 2.2. The Overexpression of AtGAP1 Promotes the Accumulation of Cell-Wall-Modifying Proteins

**Figure 3:**
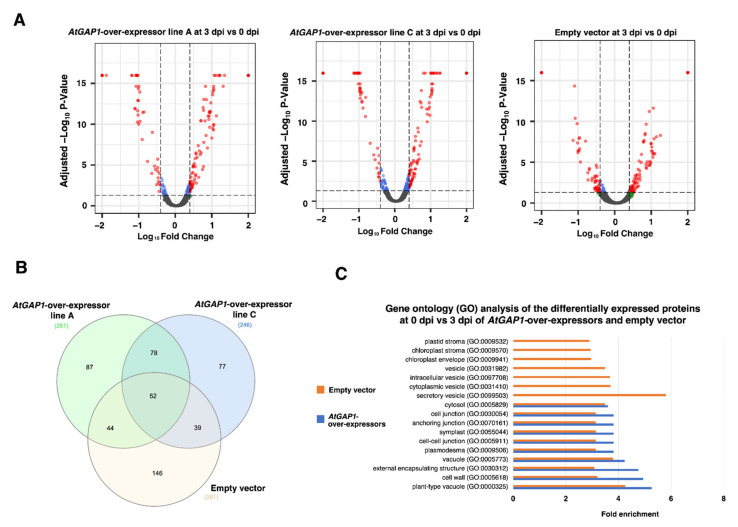
*AtGAP1*-overexpressors showed a more significant enrichment in cell-wall-related proteins in their differential protein expression profiles after the inoculation of *Pst* DC3000 than the empty vector control. The rosette leaves of five-week-old Arabidopsis plants, including the empty vector control and *AtGAP1* overexpressors (independent lines A and C) were inoculated with *Pst* DC3000. Total protein was extracted from the aerial part of the plants for protein-expression profiling. (**A**) Volcano plots showing the fold-changes in protein abundance between 3 days post-inoculation (dpi) and 0 dpi in *AtGAP1*-overexpressor line A, *AtGAP1*-overexpressor line C, and the empty vector control. The statistical significance of differential protein expression was calculated using Student’s t-test based on the default parameters in Proteome Discoverer v2.4 (Thermo Fisher Scientific, Waltham, MA, USA). The -log_10_-transformed *p*-value (Benjamini–Hochberg adjusted −log_10_ *p*-value) was plotted against log_10_-transformed protein quantity ratios for all proteins between 3 and 0 dpi in *AtGAP1*-overexpressors and the empty vector control. The volcano plot is generated via the R-based package EnhancedVolcano (ver. 1.0.1; https://github.com/kevinblighe/EnhancedVolcano (accessed on 30 April 2022) [57]). Differentially expressed protein with Benjamini–Hochberg adjusted *p*-value < 0.05 and log_10_ (|fold change|) > 0.48 (equivalent to 3-fold changes) were plotted in red. Proteins with log_10_ (|fold change|) > 0.48 but with adjusted *p*-value ≥ 0.05 were plotted in green, and proteins with log_10_ (|fold change|) ≤ 0.48 with adjusted *p*-value < 0.05 were plotted in blue. (**B**) Venn diagram showing the numbers of differentially expressed proteins after *Pst* DC3000 inoculation that were common between *AtGAP1*-overexpressors and the empty vector control. (**C**) Gene ontology (GO) analysis of the differentially expressed proteins in *AtGAP1*-overexpressing lines and the empty vector control between 3 and 0 dpi. The lists of differentially expressed proteins were compared using PANTHER from The Arabidopsis Information Resources (TAIR) database for the GO-term enrichment in cellular components. The GO terms with fold enrichment > 2 and adjusted *p*-value < 0.05 were listed. Each biological replicate was pooled from three individual plants of the same line. The results were the average of three biological replicates analyzed using Proteome Discoverer v2.4. (Thermo Fisher Scientific, Waltham, MA, USA).

The protein profiles of Arabidopsis plants inoculated with *Pst* DC3000 were studied using mass spectrometry-based label-free quantification (LFQ). The profiles from three biological replicates of each Arabidopsis line were subjected to principal component analysis (PCA) for clustering samples having similar variation characteristics (Figure S5) [56]. The differential protein expression profiles were obtained between 3 and 0 dpi in the empty vector control, and *AtGAP1-*overexpressor lines A and C, respectively (Figure 3), and represented by volcano plots (Figure 3A). In the empty vector control, 281 proteins were found to be differentially expressed between 3 and 0 dpi, whereas in *AtGAP1-*overexpressor lines A and C, 261 and 264 proteins, respectively, were found to be differentially expressed between 3 and 0 dpi (Figure 3B, Tables S1–S3). There were 109 common differentially expressed proteins between *AtGAP1*-overexpressor lines A and C (Figure 3B) showing similar expression trends. Among them, 73 were upregulated while 36 were downregulated at 3 dpi compared to day 0 of the inoculation. Among the 281 differentially expressed proteins found in the empty vector control between 3 dpi and 0 dpi, 132 proteins were upregulated at 3 dpi while 149 proteins were downregulated at 3 dpi. The differentially expressed proteins having similar expression trends in line A and line C, and those in the empty vector control, were subjected to gene ontology (GO) analysis (Figure 3C). Compared to the *AtGAP1*-overexpressors, the differentially expressed proteins in the empty vector control were more diverse in terms of cellular components (Figure 3C). For the GO terms enriched in both the empty vector control and the *AtGAP1*-overexpressors, including cytosol (GO:0005829), cell junction (GO:0030054), anchoring junction (GO:0070161), symplast (GO:0055044), cell–cell junction (GO:0005911), plasmodesma (GO:0009506), vacuole (GO:0005773), external encapsulating structure (GO:0030312), cell wall (GO:0005618), and plant-type vacuole (GO:0000325), the *AtGAP1*-overexpressors showed higher folds of enrichment in all these GO terms compared to the empty vector control (Figure 3C). The GO enrichment analysis on biological process was also conducted but defense-response-related enrichments were not observed (Figure S6).

**Figure 4:**
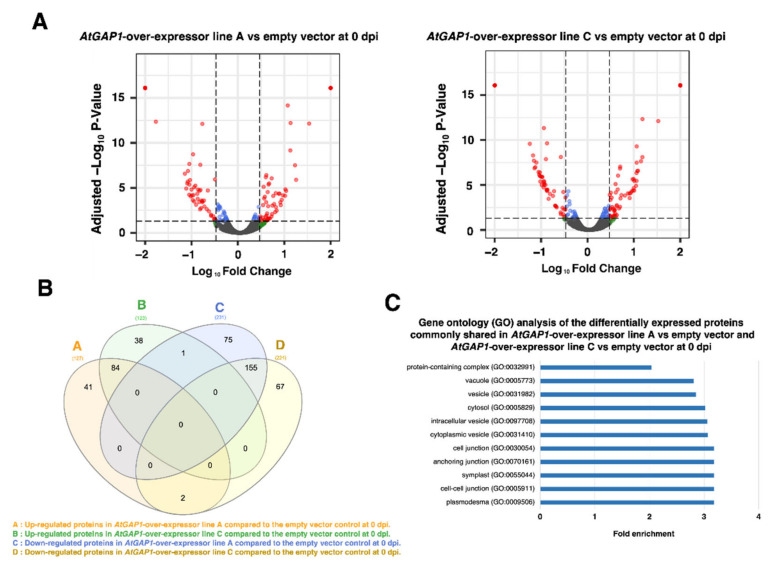
Differential protein expression profiles due to *AtGAP1*-overexpression in Arabidopsis at Day 0 of *Pst* DC3000 inoculation analyzed by liquid chromatography-mass spectrometry (LC-MS) and label-free quantification (LFQ). (**A**) Volcano plots showing the fold changes in proteins in *AtGAP1*-overexpressor A and *AtGAP1*-overexpressor C compared to the empty vector control at Day 0 of *Pst* DC3000 inoculation (0 dpi). The data from LFQ were subjected to Student’s t-test based on the default parameters in Proteome Discoverer v2.4 (Thermo Fisher Scientific, Waltham, MA, USA). Differentially expressed proteins with Benjamini–Hochberg adjusted *p*-value < 0.05 and log_10_ (|fold-change|) > 0.48 (equivalent to 3-fold changes) were plotted in red. Proteins with log_10_ (|fold change|) > 0.48 but with adjusted *p*-value ≥ 0.05 were plotted in green, and those with log_10_ (|fold change|) ≤ 0.48 with adjusted *p*-value < 0.05 were plotted in blue. (**B**) Venn diagram showing the numbers of differentially expressed proteins common between *AtGAP1*-overexpressor A and *AtGAP1*-overexpressor C when compared to the empty vector control at 0 dpi. (**C**) Gene ontology (GO) analysis of the 239 differentially expressed proteins having similar expression trends in both *AtGAP1*-overexpressors A and C compared to the empty vector control at 0 dpi. The differentially expressed proteins were classified using PANTHER from The Arabidopsis Information Resources (TAIR) database for the GO-term enrichment in cellular components. GO-terms with fold enrichment > 2 and Bonferroni-corrected *p* < 0.05 were listed. The results were the average of three biological replicates analyzed using Proteome Discoverer v2.4 (Thermo Fisher Scientific, Waltham, MA, USA). For each biological replicate, three plants were collected for protein extraction and analysis.

We also compared the protein profiles between the *AtGAP1*-overexpressing lines and the empty vector control at 0 dpi (Figure 4). The differential expressions are shown by volcano plots (Figure 4A). At 0 dpi, 242 proteins were found to be differentially expressed in both *AtGAP1*-overexpressor line A and line C compared to the empty vector control. (Figure 4). Among the 242 proteins, 239 proteins were found to have consistent differential expression trends in both *AtGAP1*-overexpressor line A and line C compared to the empty vector control (Table S4). Among the 239 proteins, 84 proteins were upregulated while 155 proteins were downregulated in the *AtGAP1*-overexpressing lines compared to the empty vector control (Table S4). These 239 proteins were subjected to GO enrichment analysis and classified into categories including protein-containing complex (GO:0032991), vacuole (GO:0005773), vesicle (GO:0031982), cytosol (GO:0005829), intracellular vesicle (GO:0097708), cytoplasmic vesicle (GO:0031410), cell junction (GO:0030054), anchoring junction (GO:0070161), symplast (GO:0055044), cell–cell junction (GO:0005911), and plasmodesma (GO:0009506) (Figure 4C). For GO enrichment analysis on the biological process, GO terms related to stress or defense responses could not be enriched with the differentially expressed proteins commonly found in *AtGAP1*-overexpressor line A and line C compared to the empty vector control at 0 dpi (Figure S7A). We therefore focused on the proteins involved in regulating the cell–cell junctions, as hinted at by the GO term analysis shown in Figure 3 and Figure 4.

**Figure 5:**
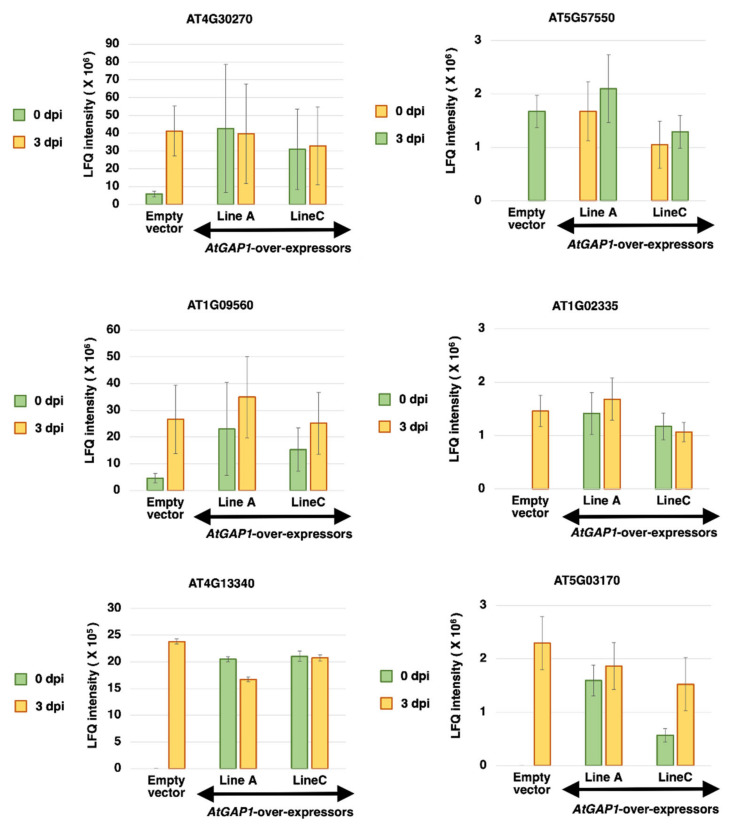
*AtGAP1* overexpression primes the production of cell-wall-modifying proteins in Arabidopsis before any exposure to the pathogen *Pst* DC3000. The label-free quantification (LFQ) intensities of cell-wall-modifying proteins extracted from the leaves of five-week-old Arabidopsis plants, including XTH24 (AT4G30270), GLP4 (AT1G09560), LRX3 (AT4G13340), GL22 (AT1G02335), XTH25 (AT5G57550), GL22 (AT1G02335), and FLA11 (AT5G03170), were compared among the empty vector control, *AtGAP1-*overexpressing line A, and *AtGAP1*-overexpressing line C at 0 and 3 dpi (days post-inoculation) of *Pst* DC3000. The error bar represents the standard error of six technical replicates from three biological replicates.

The above results suggest the regulations of proteins related to cell–cell junctions due to the overexpression of AtGAP1 (Figure 3 and Figure 4). Since cell-wall proteins are directly related to the first line of defense against pathogens [58], we investigated in greater detail the abundance of individual cell-wall-modifying proteins (Figure 5). Cell-wall-modifying proteins, including xyloglucan endotransglucosylase/hydrolase 24 (XTH24, AT4G30270), germin-like protein 4 (GLP4, AT1G09560), leucine-rich repeat extensin-like protein 3 (LRX3, AT4G13340), XTH25 (AT5G57550), germin-like 22 (GL22, AT1G02335), and fasciclin-like arabinogalactan 11 (FLA11, AT5G03170), were found to be induced at 3 dpi in the empty vector control (Figure 5). Interestingly, these proteins were already more abundant in both *AtGAP1*-overexpressing lines A and C than in the empty vector control at 0 dpi (Figure 4). In the *AtGAP1*-overexpressor lines A and C, the levels of some of these proteins were further induced at 3 dpi (Figure 5).

### 2.3. AtGAP1 Enhances Mesophyll Cell Wall Thickness

**Figure 6:**
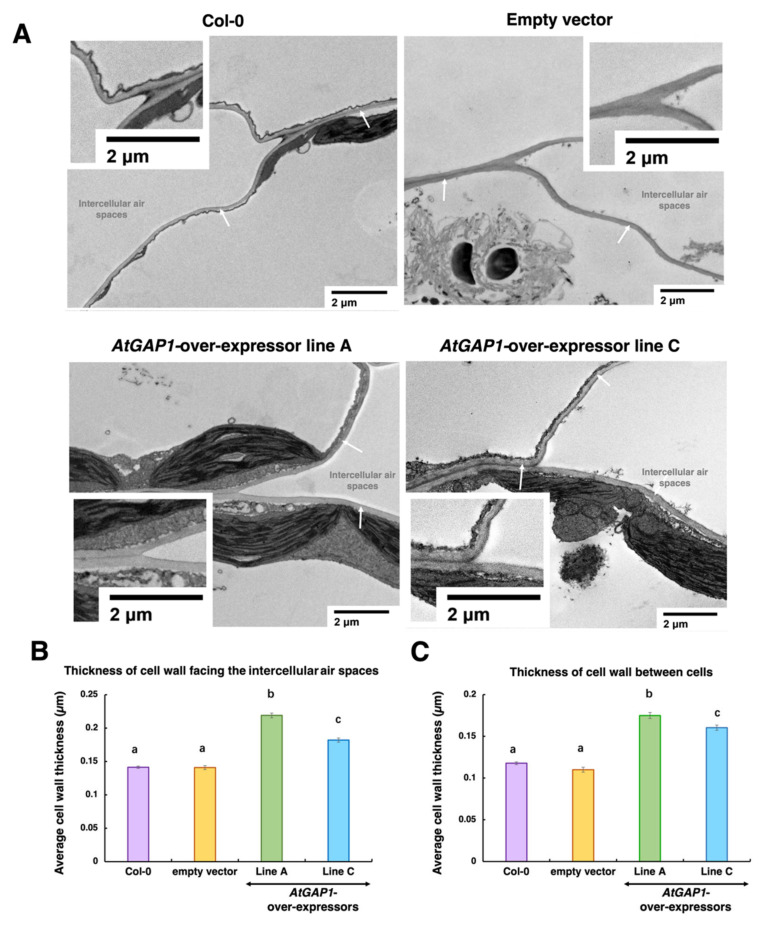
*AtGAP1* enhances the cell-wall thickness of mesophyll cells. (**A**) Representative transmission electron microscopy (TEM) images of the cross-sections of four-week-old rosette leaf cells of untreated Arabidopsis plants, including wild type (Col-0), empty vector control, and *AtGAP1*-overexpressor lines A and C. Cell-wall structures were indicated by white arrows. Scale bar: 2 μm. (**B**) The thickness of the cell wall facing the intercellular air spaces was measured for ≥50 cells from ≥20 fields. (**C**) The thickness of the cell wall that was in contact with another cell was measured from ≥20 cells from ≥10 fields. For each cell, the thickness was determined by averaging the thickness of five random points along the cell wall. Different letters above the bars indicate significant differences at *p* < 0.05, analyzed using one-way ANOVA followed by post-hoc Tukey honest significant difference (HSD) test. Error bar represents the standard error of all the measured cells.

The protein profiling results suggested the regulation of cell-wall thickness by AtGAP1 to achieve the enhanced resistance to *Pst* DC3000 in Arabidopsis. To validate the effects of *AtGAP1*-overexpression on the cell wall, cross-sections of rosette leaves from four-week-old untreated Arabidopsis plants, including wild type, empty vector control, and *AtGAP1-*overexpressor lines A and C, were examined using transmission electron microscopy (TEM) to determine the cell-wall thickness (Figure 6). Compared to the wild type and empty vector control, the mesophyll cells of *AtGAP1-*overexpressors had significantly thicker cell walls in regions facing intercellular air space and regions where two cells are in contact (Figure 6). TEM images in a broader view are shown in Supplementary Figure S8.

### 2.4. AtGAP1 Reduces Stomatal Aperture with the Effect Being Reversible by Additional GTP

**Figure 7:**
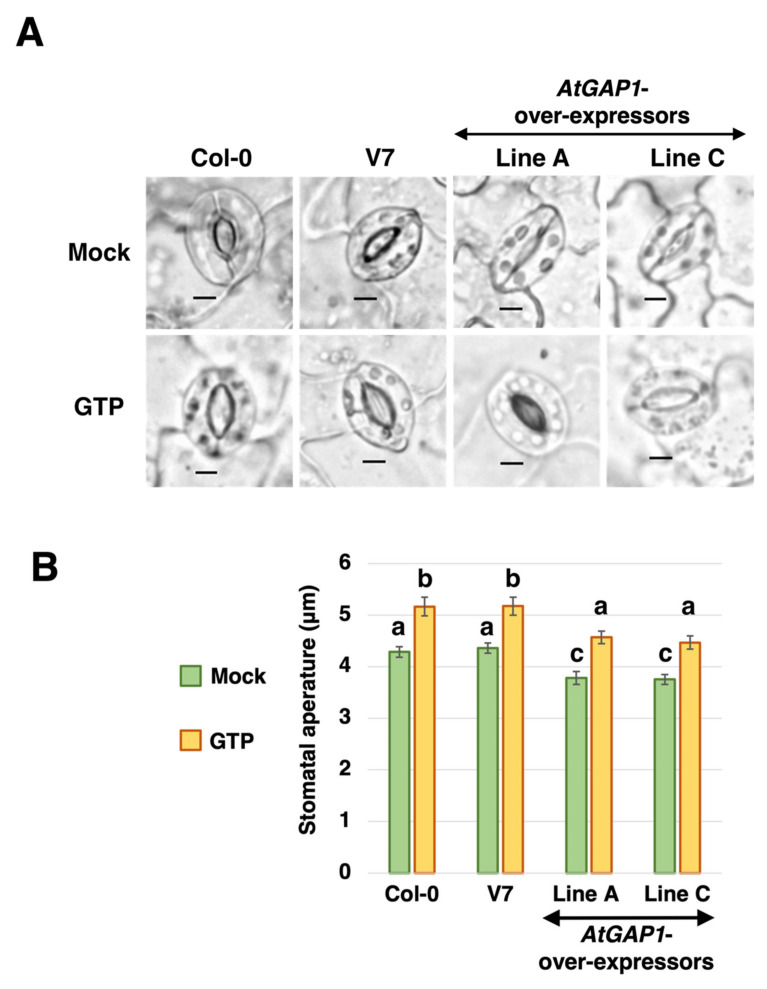
*AtGAP1*-overexpression reduces stomatal aperture in Arabidopsis with the effect being reversible by additional GTP. Detached rosette leaves of four-week-old Arabidopsis plants, including wild type, empty vector control (V7), and *AtGAP1*-overexpressor lines A and C, were treated with or without (mock) 200 μM GTP under light for 2 h. (**A**) Representative images of guard cells observed using a light microscope. Scale bar: 5 μm. (**B**) Stomatal apertures of wild type, empty vector control (V7), and *AtGAP1*-overexpressor lines A and C treated with or without (mock) 200 μM GTP. For each line, the stomatal apertures of ≥25 pairs of guard cells were measured. Error bar: standard error of all the cells measured. A similar trend was observed in another biological replicate (Figure S9). Different letters above the bars indicate significant differences at *p* < 0.05, analyzed using one-way ANOVA followed by post-hoc Tukey honestly significant difference (HSD) test.

Besides the cell wall, stomatal aperture is also an important structural feature in restricting the entry of pathogens [59,60]. The rosette leaves of four-week-old Arabidopsis plants, including wild type (Col-0), empty vector control, and *AtGAP1*-overexpressing lines A and C, were subjected to stomatal aperture analyses. Results showed that the overexpression of *AtGAP1* reduced the stomatal aperture (Figure 7). When treated with 200 μM GTP, the stomatal aperture in all lines increased significantly compared to the mock treatment, and the stomatal apertures in the GTP-treated *AtGAP1*-overexpressing lines were comparable to those in the wild type or the empty vector control with mock treatment (Figure 7). It appears that AtGAP1 promotes pathogen resistance by reducing stomatal aperture.

## 3. Discussion

Previous studies suggested the role of OsGAP1, the homolog of AtGAP1, in promoting the resistance to pathogens [20], and the role of AtGAP1 (CAR4) in regulating ABA signaling [35,36]. However, the role of AtGAP1 in regulating the resistance to pathogens remained unclear. In this study, we showed that the expression of *AtGAP1* is inducible by *Pst* DC3000 inoculation. At 0 dpi, compared to the wild type and the empty vector control, the overexpression of *AtGAP1* resulted in the increased expression level of *PR1,* which is known to be induced by *Pst* DC3000 and other systemic acquired response (SAR) inducers including 2,6-dichloroisonicotinic acid (INA) and benzo(1,2,3)thiadiazole-7-carbothioic acid *S*-methyl ester (BTH) [61]. The Arabidopsis mutant, cep, which constitutively expresses *PR1*, has enhanced resistance to pathogens, including *Pseudomonas syringae* pv. *maculicola* and *Peronospora parasitica* isolate EMWA [62]. The increased expression of *PR1* and the enhanced resistance to *Pst* DC3000 inoculation due to *AtGAP1* overexpression (Figure 1) are consistent with the previous reports showing the positive correlations between *PR1* expression level and pathogen resistance in Arabidopsis. 

In the proteomic analysis, defense-response-related and stomatal-movement-related enrichments using GO enrichment analysis were not observed. It is possible that the throughput of the mass-spectrometry-based analysis was not high enough to have a broad coverage of all the proteins. In addition, to ensure the confidence of the analysis, only proteins showing the same expression trends in both *AtGAP1*-overexpressing line A and line C compared to the empty vector control were subjected to GO enrichment analysis. Therefore, the amount of proteins subjected to GO enrichment analysis was further reduced but the confidence of the analyses was upheld. Since the proteomic analyses confidently pointed to the regulation of proteins related to the cell–cell junction (Figure 3 and Figure 4), in the subsequent analysis, we narrowed down the investigation to this category and focused on proteins related to the formation of the cell wall, which is an important barrier for restricting the entry of pathogens [47,48,49].

Cell-wall-modifying proteins, including XTH24 (AT4G30270), GLP4 (AT1G09560), LRX3 (AT4G13340), XTH25 (AT5G57550), GL22 (AT1G02335), and FLA11 (AT5G03170), were more abundant in *AtGAP1-*overexpressors at 0 dpi compared to the empty vector control (Figure 5). XTH is a class of cell-wall-modifying enzymes with endotransglucosylase or hydrolase activity [63,64]. AtXTH3 catalyzes the cross-link between xyloglucan and cellulose to form insoluble material that is potentially involved in cell-wall formation [65]. Besides this, the overexpression of *AtXTH18*, *AtXTH19*, or *AtXTH20* in Arabidopsis enhanced the mechanical strength of the cell wall [66]. Similarly, the ectopic expression of *SkXTH1* from *Selaginella kraussiana* in onion led to the deposition of more cell-wall material [67]. The ectopic expression of *OsGLP1* in tobacco was shown to enhance the cross-linking of cell-wall components [68]. On the other hand, the siRNA-mediated silencing of *OsGLP1* led to the enhanced susceptibility of rice plants to fungal infection [69]. Under *AtGAP1* overexpression, the enhanced accumulation of these cell-wall-modifying proteins (Figure 5) and the enhanced tolerance to *Pst* DC3000 (Figure 2) are consistent with previous findings. The enhanced thickness of the mesophyll cell wall due to *AtGAP1* overexpression (Figure 6) is in line with the accumulation of cell-wall-modifying proteins (Figure 5) and the enhanced tolerance to *Pst* DC3000 (Figure 2). 

We also investigated the effect of *AtGAP1* overexpression on stomatal aperture, which is another means of restricting the entry of foliar pathogens [40]. The overexpression of *AtGAP1* led to a reduced stomatal aperture compared to the wild type and empty vector control (Figure 7). This, in combination with the enhanced mesophyll cell-wall thickness (Figure 6), forms an increased barrier against pathogen entry into the leaf cells.

Since AtGAP1 (CAR4) is a positive regulator of ABA signaling [35,36], it is speculated that AtGAP1 regulates the thickness of mesophyll cell wall by regulating ABA sensitivity. In Arabidopsis, a previous study reported that the prohibition of ABA synthesis would interfere with secondary cell-wall thickness and lignification [70]. However, the role of ABA in regulating the primary cell wall, which is the type of cell wall existing in the mesophyll cells of four-week-old rosette leaves, remains unclear. On the other hand, OsGAP1, a close homolog of AtGAP1, was demonstrated to activate the GTPase activity of AtYchF1 [9,20,21]. It is also speculated that the effect of AtGAP1 on the mesophyll cell-wall thickness is associated with the modulation of cellular GTPase activity. Previous studies have suggested the positive roles of GTPases in the formation of primary cell wall [33,34]. The positive role of AtGAP1 in enhancing the mesophyll cell-wall thickness observed in this study is consistent with previous reports. The effect of AtGAP1 on regulating cellular GTPase activity is also supported by the stomatal aperture test. When treated with GTP, the effect on stomatal aperture brought forth by *AtGAP1* overexpression was reversed (Figure 7). GTP treatment was previously demonstrated to induce stomatal opening in detached Arabidopsis leaves [71]. The observation of the complemented effect on stomatal aperture by additional GTP suggests that AtGAP1 may reduce stomatal aperture by activating GTPase activities, and therefore decreasing the store of GTP available for other enzyme activities related to maintaining stomatal aperture. On the other hand, ABA is the major hormone that mediates stomatal closure [72]. The steady-state stomatal aperture of Arabidopsis was also revealed to be regulated by ABA [73]. It is, therefore, possible that the reduced stomatal aperture under *AtGAP1* overexpression was also a result of the enhanced ABA signaling. 

## 4. Materials and Methods

### 4.1. Plant Materials, Growth Condition, and Pathogen Inoculation of Arabidopsis

Arabidopsis plants, including the wild type (Col-0), empty vector control (V7), independent *AtGAP1*-overexpressor line A and line C [21], were grown on Floragard potting soil in a growth chamber with the following settings: 22–24 °C; light intensity 80–120 μE with a 16 h light:8 h dark cycle; relative humidity 70–80%. *Pseudomonas syringae* pv. *tomato* DC3000 (*Pst* DC3000) was used as the pathogen for inoculation into the rosette leaves of five-week-old Arabidopsis plants using the protocol in the previous reports [21,74]. The pathogen titer was determined in samples at 0 and 3 d after inoculation (dpi) using a plate count method [21,74]. After pathogen inoculation, the aerial part of the Arabidopsis plant was harvested and frozen in liquid nitrogen prior to total RNA extraction.

### 4.2. RNA Extraction, cDNA Synthesis, and Gene Expression Analysis

The frozen plant materials were pulverized using pestle and mortar. Total RNA was extracted using Trizol^TM^ Reagent (Cat.#15596018, Thermo Fisher Scientific, Waltham, MA, USA) according to the manufacturer’s instructions. Tissues from three individual Arabidopsis plants were pooled as one biological replicate. The RNA was then treated with DNase I (Cat.#18068015, Thermo Fisher Scientific, Waltham, MA, USA) prior to cDNA synthesis. cDNA was generated using a High-Capacity cDNA Reverse Transcription Kit with RNase Inhibitor (Cat.#4374966, Thermo Fisher Scientific, Waltham, MA, USA) according to the manufacturer’s protocol with the random primers being replaced by oligo(dT)_20_ to make up a final concentration of oligo(dT)_20_ in 20 μM. The cDNA was subjected to expression analysis by quantitative reverse transcription PCR (RT-qPCR) with the use of SsoAdvanced Universal SYBR Green Supermix (Cat.#1725270, Bio-Rad, Hercules, CA, USA) according to the manufacturer’s protocol. *ACT7* (AT5G09810) (Czechowski 2005) was used as the reference gene for normalization. The relative gene expression was calculated using the 2^−ΔΔCT^ method [55]. Primers used for qPCR are listed in Table S6.

### 4.3. Protein Extraction and Protein Profile Analysis by Liquid Chromatography-Tandem Mass Spectrometry (LC-MS/MS)

Total proteins were extracted from the aerial part of five-week-old plant according to the protocol described previously [75] with minor modifications. Samples from three biological replicates were collected. Three individual Arabidopsis plants were pooled as one biological replicate. Proteins from three biological replicates were analyzed independently for protein identification using LC-MS/MS. After the *Pst* DC3000 inoculation, the aerial part of the Arabidopsis plant was frozen in liquid nitrogen and then pulverized using pestle and mortar. Around 100 mg of plant powder was resuspended in five volumes of total protein extraction buffer [290 mM sucrose, 250 mM Tris (pH 7.6), 25 mM EDTA (pH 8.0), 10 mM KCl, 25 mM NaF, 50 mM Na pyrophosphate, 1 mM ammonium molybdate, 1 mM PMSF, 1X Halt™ Protease Inhibitor Cocktail (Cat.#78430, Thermo Fisher Scientific, Waltham, MA, USA)]. Homogenized samples were centrifuged at 10,000× *g* for 5 min at 4 °C to remove the cell debris. Proteins were precipitated from the extract using chloroform/methanol precipitation. The protein pellet was then dried on ice and immediately lyzed with five volumes of lysis buffer (*w*/*v*) [(8 M urea, 50 mM Tris-HCl (pH 8.0), 30 mM NaCl, 1 mM CaCl2, 20 mM sodium butyrate, 1X Halt™ Protease Inhibitor Cocktail (Cat.#78430, Thermo Fisher Scientific, Waltham, MA, USA)]. The protein concentration was determined using Pierce^TM^ BCA Protein Assay Kit (Cat.#23225, Thermo Fisher Scientific, Waltham, MA, USA). Ten micrograms of protein were treated with 5 mM DTT at 37 °C for 30 min, followed by 20 mM iodoacetamide at room temperature for 30 min and then 5 mM DTT at 37 °C for 30 min. One-twentieth (*w*/*w*) of the protein amount was added according to the manufacturer’s instruction for digestion at 37 °C overnight. The digested peptides were desalted with a Pierce^TM^ C18 Spin Column (Cat.#89873, Thermo Fisher Scientific, Waltham, MA, USA) for later analyses.

The desalted peptides were resuspended with 0.1% formic acid in ultrapure water. Five hundred nanograms of desalted peptides were injected into the LC Ultimate 3000 RSLCnano system equipped with a C-18 μ-precolumn (300-μm i.d. × 5 mm) with an Acclaim Pepmap RSLC nanoViper C-18 column (75 μm × 25 cm) coupled to the Orbitrap Fusion Lumos Tribrid mass spectrometer (Thermo Fisher Scientific, Waltham, MA, USA). A mixture of ultrapure water with 1.9% acetonitrile and 0.1% formic acid was used as mobile phase A while acetonitrile with 2% ultrapure water and 0.1% formic acid was used as mobile phase B in the liquid chromatography (LC). The peptide samples were separated against the gradient profile with a 50 °C chamber at a flow rate of 300 nL min^−1^. The setting of the gradient profile was as follows: 0–5 min 0% mobile phase B; 5–8 min 0–6% mobile phase B; 8–48 min, 6–18% mobile phase B; 48–58 min, 18–30% mobile phase B; 58–65 min, 30–80% mobile phase B; 65–75 min, 0% mobile phase B. Each desalted peptide sample was analyzed twice to eliminate the instrumental variations.

The nanoLC/MS was controlled using Xcalibur software (Thermo Fisher Scientific, Waltham, MA, USA). The MS/MS identification using Proteome Discoverer v2.4 (Thermo Fisher Scientific, Waltham, MA, USA) against the Arabidopsis protein database (TAIR10) with the built-in SEQUEST HT program. The MS precursor mass tolerance was set to 10 ppm, fragment mass tolerance of 0.02 Da, two missed trypsin cleavage in maximum, dynamic cysteine carbamidomethylation (+57.021 Da), methionine oxidation (+15.995 Da), and N-terminal protein acetylation (+42.011 Da). All search results at the peptide spectrum level were subsequently validated by the built-in Percolator program and accepted at a false discovery rate (FDR) with a q-value ≤ 0.01. Samples were compared using the label-free quantification (LFQ) method according to the protocol in Proteome Discoverer v2.4. Only proteins appearing in at least two biological replicates with a three-fold difference in abundance with adjusted *p*-value < 0.05 (using the Benjamini–Hochberg correction) in each comparison pair were used for gene ontology (GO) enrichment analyses filtered with adjusted *p*-value < 0.05 after Bonferroni correction for multiple testing [76].

### 4.4. Transmission Electron Microscopy (TEM)

TEM sample preparation was done following a protocol previously described, with minor modifications [77,78]. Rosette leaves from three four-week-old Arabidopsis plants from each line were cut to obtain a 1 mm × 5 mm section with a blade. The sections were then fixed with 2.5% glutaraldehyde (Cat.#G7776, Merck) in 0.1 M sodium phosphate buffer (PBS, pH7.2) at room temperature for 4 h. The samples were then washed with 0.1 M PBS (pH7.2) for 10 min twice. Samples were post-fixed with 1% osmium tetroxide (OsO_4_) in PBS for 2 h at room temperature with two subsequent 10-min washes using 0.1 M PBS (pH7.2). The fixed samples were then dehydrated using the following ethanol (EtOH) gradient: 50% EtOH, 70% EtOH, 85% EtOH, 95% EtOH twice, and 100% EtOH thrice. Each wash was performed for 10 min. The dehydrated samples were infiltrated with a series of ethanol:Spurr’s resin mixtures (*v*:*v*) at room temperature: 2:1 for 4 h, 1:1 for 4 h, 1:2 overnight, and then pure Spurr’s resin for 3 h. Samples were then embedded in pure Spurr’s resin for 16 h at 68 °C. The embedded samples were then trimmed using a blade before being sectioned using an EM UC7 Ultramicrotome (Leica, Germany) to a 70-nm thickness. The sections were then mounted onto 200-mesh copper grids (Cat.#EMS200-Cu, Electron Microscope Sciences). The samples were then stained with 1% uranyl acetate, followed by 0.5% lead citrate, and then observed under an electron microscope (Model H-7650, Hitachi, Japan) at 80 kV.

### 4.5. Stomatal Aperture Test

The rosette leaves of four-week-old Arabidopsis plants were detached for the stomatal aperture test. The detached leaves were pre-treated in a perfusion solution (50 mM KCl, 10 mM MES, pH 7.0) under light for 2 h before being treated with or without GTP (200 μM) in the perfusion solution (50 mM KCl, 10 mM MES, pH 7.0) under light for 2 h. After the treatment, the lower epidermis was peeled off to observe the stomatal aperture using a light microscope (Model DM2000, Leica, Wetzlar, Germany) equipped with a digital camera (FLEXACAM-C1-5020240068). The images were then analyzed using Leica Application Suite X (LAS X) to measure the stomatal aperture. The stomatal aperture test was done in two biological replicates. In each biological replicate, the stomatal apertures of ≥25 pairs of guard cells from each Arabidopsis line were measured.

## 5. Conclusions

In this study, we generated *AtGAP1*-overexpressing Arabidopsis lines and showed that the overexpression of *AtGAP1* promoted the resistance of the plants against the pathogen, *Pst* DC3000. Mass-spectrometry-based LFQ proteomic analyses hinted that AtGAP1 enhances disease resistance via promoting the accumulation of cell-wall-modifying proteins. Electron microscopy and stomatal aperture test results strongly suggest that AtGAP1 enhances the disease resistance of the plants by increasing mesophyll cell-wall thickness and reducing stomatal aperture before any contact with pathogens. Previous research has suggested that OsGAP1 [20], the rice homolog of AtGAP1, is a positive regulator of pathogen resistance. AtGAP1 was also demonstrated to be a positive regulator of ABA signaling [35,36]. In this study, we revealed an additional function of AtGAP1 as a positive regulator of pathogen resistance by promoting mesophyll cell-wall thickness and reducing stomatal aperture. Based on the experimental results and previous findings, we speculated that AtGAP1 mediates these phenotypes by regulating ABA sensitivity and cellular GTPase activity. Although the detailed mechanisms remain to be elucidated, the alteration of the abundance of cell-wall-modifying proteins and mesophyll cell-wall thickness by AtGAP1 shed light on future directions for the research on the functions of GAPs.

## Data Availability

All data supporting the reported results can be found in the main text and the supplementary files.

## Supplementary Materials

The following supporting information can be downloaded at: https://www.mdpi.com/article/10.3390/ijms23147540/s1.
