# Supplementary material for: AtGAP1 Promotes the Resistance to Pseudomonas syringae pv. tomato DC3000 by Regulating Cell-Wall Thickness and Stomatal Aperture in Arabidopsis
Source: Int J Mol Sci. 2022 Jul 7;23(14):7540. doi: 10.3390/ijms23147540 (PMC9323218; doi:10.3390/ijms23147540)
Supplement: Supplementary file 1 [file ijms-23-07540-s001.zip › Supplementary_AMP_7 July 22.pdf]

Article

AtGAP1 Promotes the Resistance to *Pseudomonas syringae* pv. *tomato* DC3000 by Regulating Cell-Wall Thickness and Stomatal Aperture in Arabidopsis

Sau-Shan Cheng, Yee-Shan Ku *, Ming-Yan Cheung and Hon-Ming Lam *

Centre for Soybean Research of the State Key Laboratory of Agrobiotechnology and School of Life Sciences, The Chinese University of Hong Kong, Hong Kong, China; chengsaushan@yahoo.com (S.-S.C.); cheungmy@cuhk.edu.hk (M.-Y.C.)

* Correspondence: ysamyku@cuhk.edu.hk (Y.-S.K.); honming@cuhk.edu.hk (H.-M.L.)

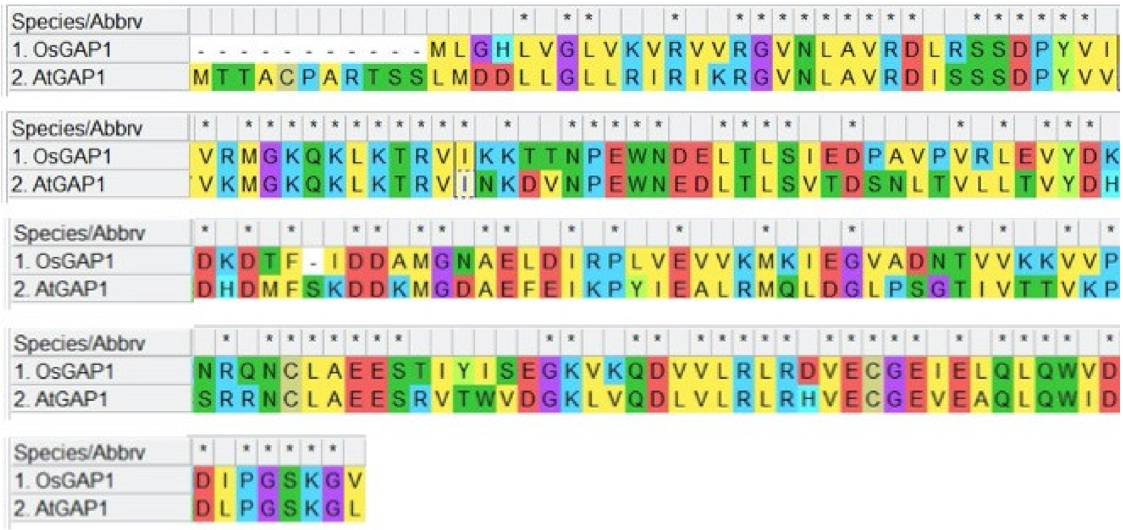

Figure S1. Alignment of the amino acid sequences of OsGAP1 (NP_001046709) and AtGAP1 (AT3G17980). The sequence alignment was done by ClustalW using MEGA11 [1]. * Identical residues. Different colors indicate amino acids of different groups.

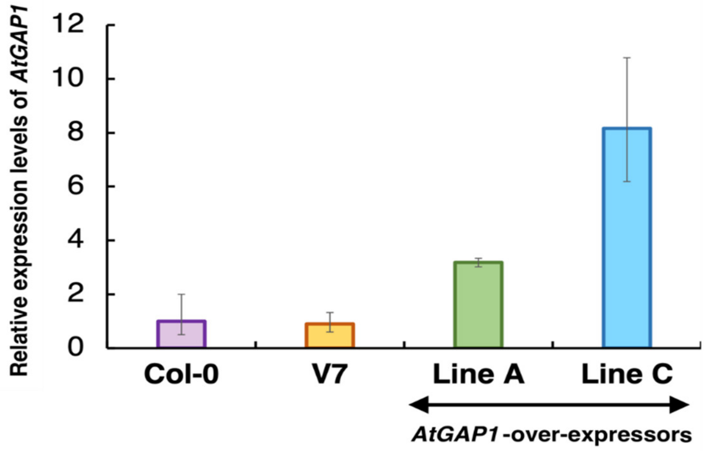

Figure S2. Detection of *AtGAP1* expression by RT-qPCR. Total RNA was extracted from the leaves of four-week-old Arabidopsis plants including wild type (Col-0), empty vector-transformed (V7), and *AtGAP1*-over-expressor lines A and C and reverse-transcribed to produce cDNA for expression

analyses. The expression level of *AtGAP1* was normalized against that in Col-0, using *ACT7* (AT5G09810) as the reference gene [2], by the $2^{-\Delta\Delta Ct}$ method [3]. The leaves from three plants of each line were pooled as one sample for total RNA extraction and expression analysis. For each sample, three technical repeats of the RT-qPCR were performed. Error bars represent the standard errors of the three technical repeats.

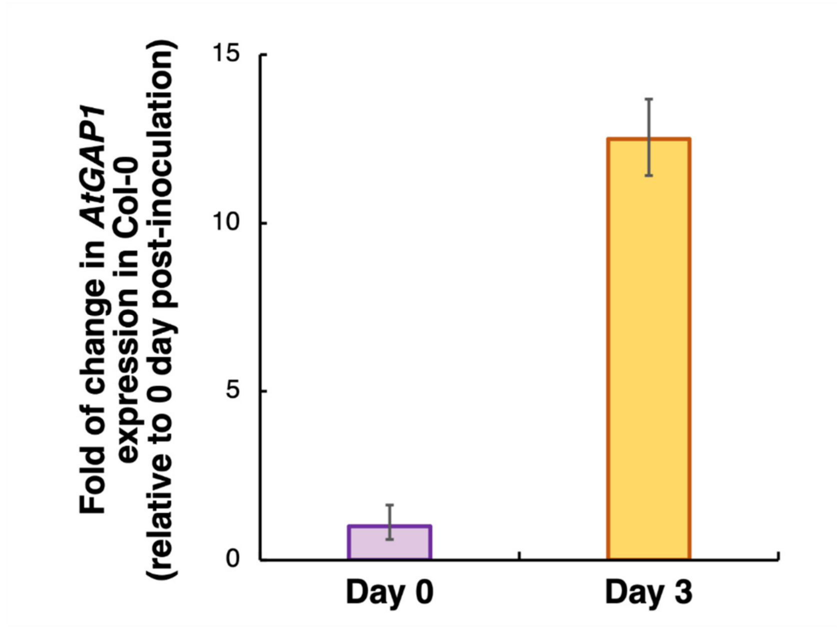

Figure S3. A second biological replicate showing the relative expressions of *AtGAP1* in the wild type Arabidopsis plant (Col-0) at Day 0 and Day 3 of *Pst* DC3000 inoculation. The expression level of *AtGAP1* at Day 3 was normalized against that at Day 0, using *ACT7* (AT5G09810) as the reference gene [2], by the $2^{-\Delta\Delta Ct}$ method [3]. Three plants of each line were pooled as one sample for total RNA extraction and expression analysis. For each sample, three technical repeats of the RT-qPCR were performed. Error bars represent the standard errors of the three technical repeats.

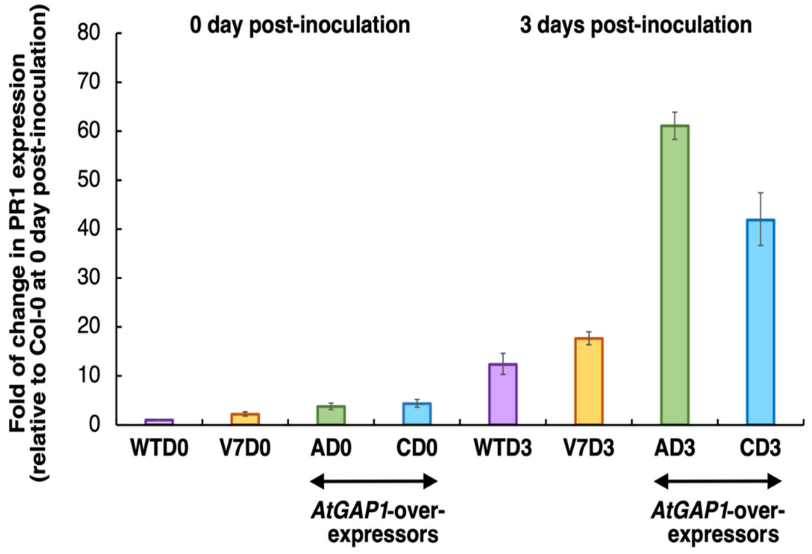

Figure S4. A second biological replicate showing the relative expressions of *PR1* in WT (Col-0), V7 (empty vector-transformed), and *AtGAP1*-over-expressors (lines A and C) at 0 d (D0) and 3 d (D3) after *Pst* DC3000 inoculation. The expression level of *PR1* was normalized against WTD0, using *ACT7* (AT5G09810) as the reference gene [2], by the $2^{-\Delta\Delta Ct}$ method [3]. Three plants of each line were pooled as one sample for total RNA extraction and expression analysis. For each sample, three technical repeats of the RT-qPCR were performed. Error bars represent the standard errors of the three technical repeats.

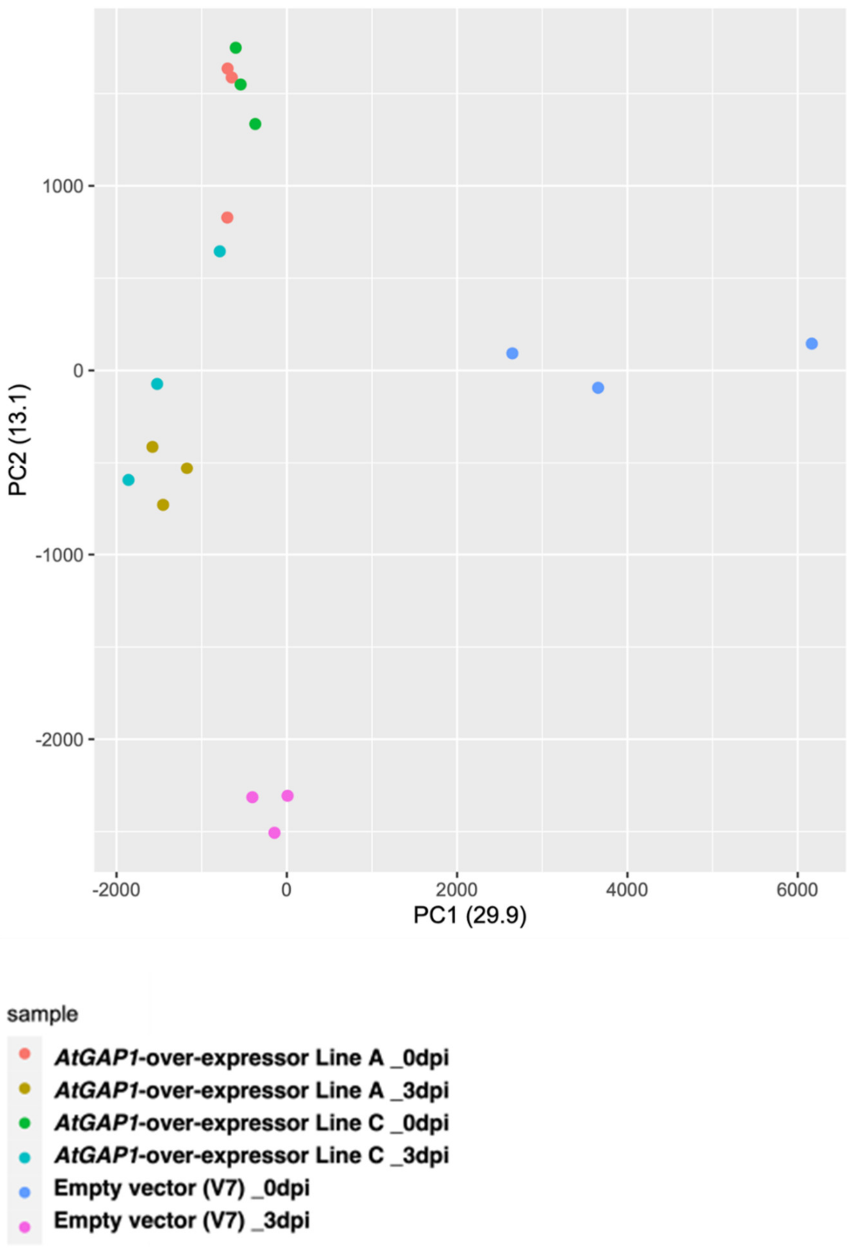

Figure S5. Principal component analysis (PCA) of protein abundance in terms of label-free quantification (LFQ) intensities. The LFQ intensities were calculated using Proteome Discoverer v2.4 (ThermoFisher Scientific, Waltham, MA, USA) against the Arabidopsis protein database (TAIR10) with the built-in SEQUEST HT program. The PCA plot was generated using R package ggplots with default settings [4]. Each dot represents one biological replicate, which was composed of three individual plants. dpi, days post-inoculation.

A

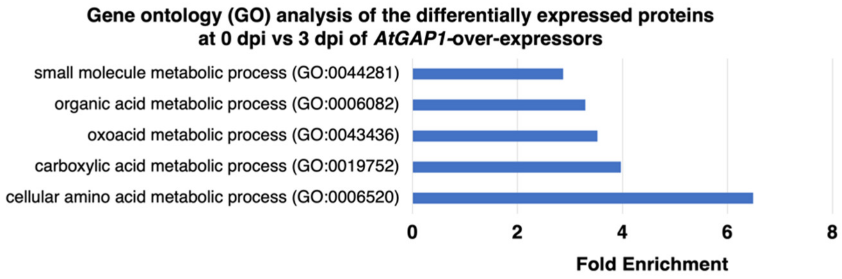

B

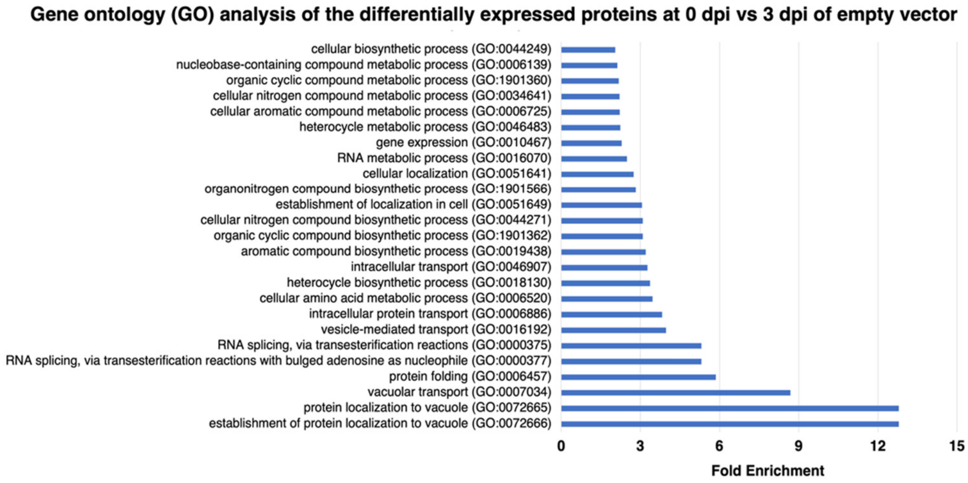

Figure S6. Gene ontology (GO) analysis on biological process of the differentially expressed protein specifically found in the comparison between **(A)** *AtGAP1*-over-expressing lines and **(B)** empty vector control at 0 and 3 dpi. The lists of differentially expressed proteins were compared using PANTHER from The Arabidopsis Information Resources (TAIR) database for the GO-term enrichment in cellular components. The GO terms with fold enrichment >2 and adjusted p-value<0.05 were listed. Each biological replicates were pooled from three individual plant of the same line. The results were the average of three biological replicates analyzed using Proteome Discoverer v2.4 (Thermo Fisher Scientific, Waltham, MA, USA).

A

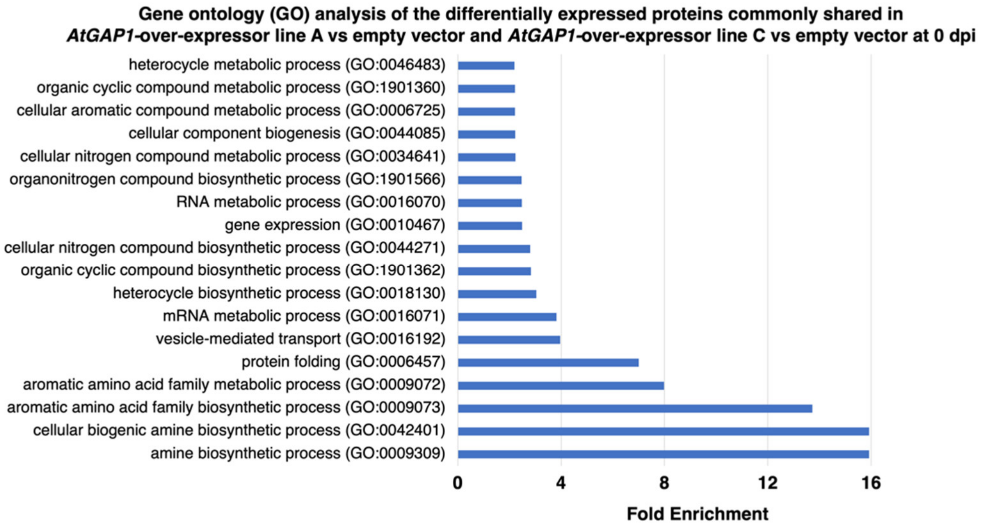

B

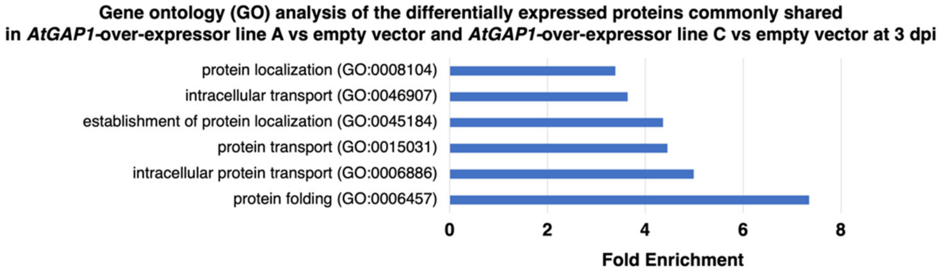

Figure S7. Gene ontology (GO) analysis on biological process of the differentially expressed protein commonly found in the comparison between *AtGAP1*-over-expressing lines and the empty vector control at 0 and 3 dpi respectively. Gene ontology (GO) analysis of the differentially expressed proteins in *AtGAP1*-over-expressing lines and the empty vector control at **(A)** 0 dpi and **(B)** 3 dpi. The lists of differentially expressed proteins were compared using PANTHER from The Arabidopsis Information Resources (TAIR) database for the GO-term enrichment in cellular components. The GO terms with fold enrichment >2 and adjusted p-value<0.05 were listed. Each biological replicates were pooled from three individual plant of the same line. The results were the average of three biological replicates analyzed using Proteome Discoverer v2.4 (Thermo Fisher Scientific, Waltham, MA, USA).

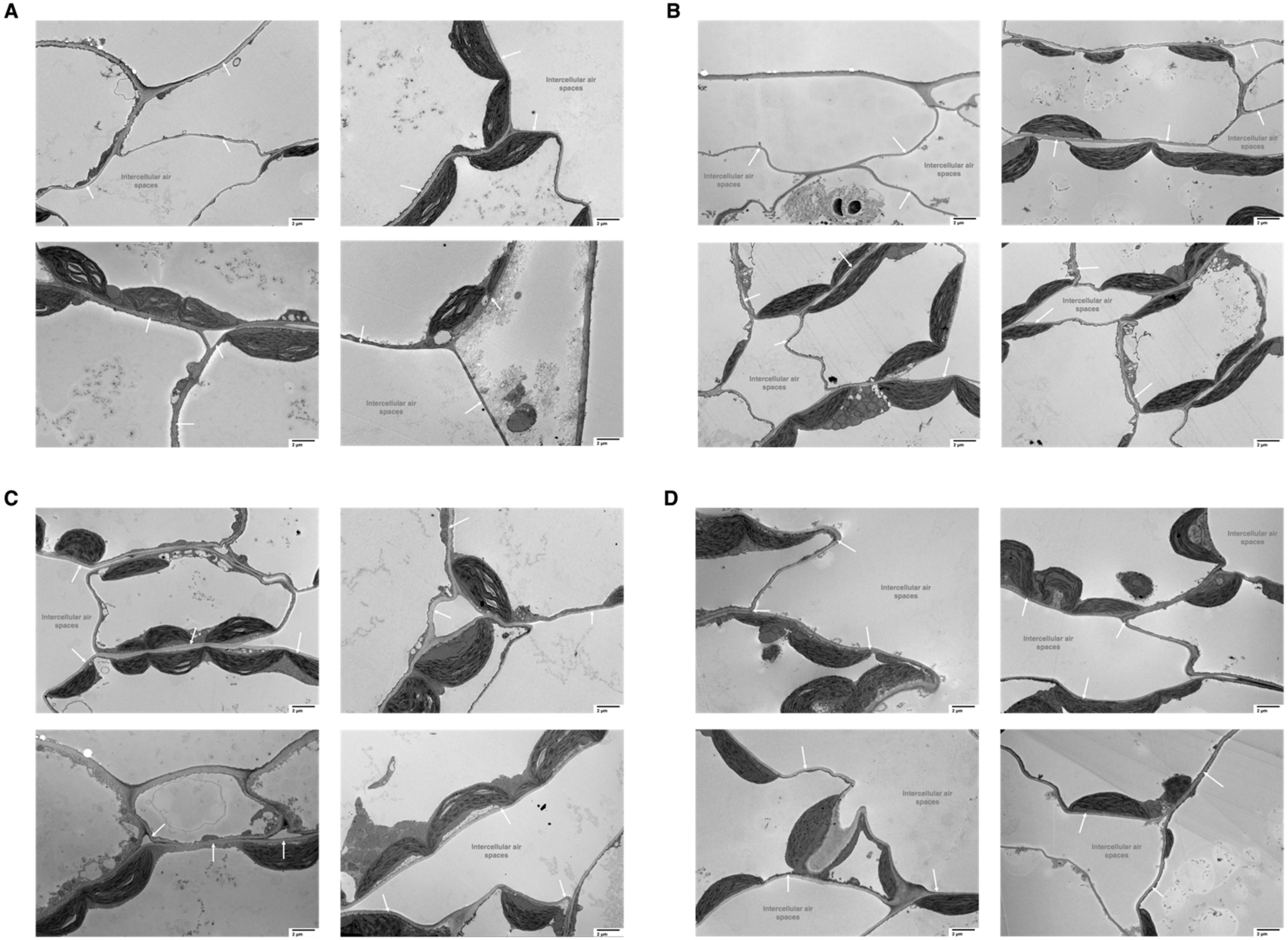

Figure S8. Transmission electron microscopy (TEM) images of the cross-sections of four-week-old rosette leaf cells of untreated Arabidopsis plants in a broad view. **(A)** Wild type (Col-0), **(B)** empty vector control, **(C)** *AtGAP1*-over-expressor line A and **(D)** *AtGAP1*-over-expressor lines C. Cell wall structures were indicated by white arrows. Scale bar: 2 μm.

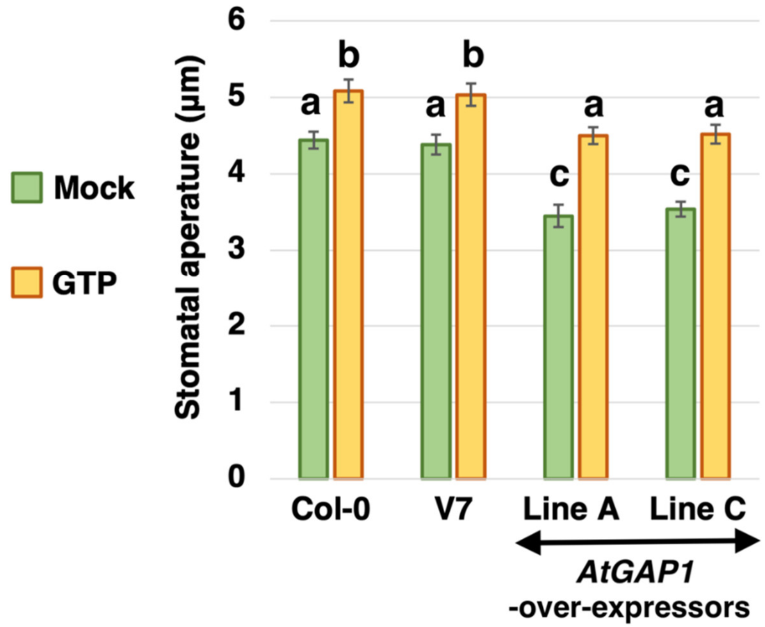

Figure S9. A second biological replicate of the stomatal aperture test. Detached rosette leaves of four-week-old Arabidopsis plants, including wild type (Col-0), empty vector control (V7), and *AtGAP1*-overexpressor lines A and C, were treated with 200 µM GTP or without (mock) under light for 2 h. For each line, the stomatal apertures of ≥ 25 pairs of guard cells were measured. Error bar: standard error. Different letters above the bars indicate significant differences at $P < 0.05$, analyzed using one-way ANOVA followed by post-hoc Tukey honestly significant difference (HSD) test.

References

1. Tamura, K.; Stecher, G.; Kumar, S. MEGA11: Molecular evolutionary genetics analysis version 11. Mol. Biol. Evol. 2021, 38, 3022–3027.
2. Czechowski, T.; Stitt, M.; Altmann, T.; Udvardi, M.K.; Scheible, W.-R. Genome-wide identification and teseting of superior refernece genes for transcript normalization in Arabidopsis. Plant Physiol. 2005, 139, 5–17.
3. Livak, K.J.; Schmittgen, T.D. Analysis of relative gene expression data using real-time quantitative PCR and the 2(-Delta Delta C(T)) method. Methods 2001, 25, 402–408.
4. Wickham, H. Manipulating data In ggplot2: Elegant Graphics for Data Analysis. In; Wickham, H., Ed.; Springer New York: New York, NY, 2009; pp. 157–175 ISBN 978-0-387-98141-3.
